# CD4+ T Cell Immune Specificity Changes After Vaccination in Healthy And COVID-19 Convalescent Subjects

**DOI:** 10.3389/fimmu.2021.755891

**Published:** 2022-01-19

**Authors:** Laura Esparcia-Pinedo, Pedro Martínez-Fleta, Noelia Ropero, Paula Vera-Tomé, Hugh T. Reyburn, José M. Casasnovas, José M. Rodríguez Frade, Mar Valés-Gómez, Carlos Vilches, Enrique Martín-Gayo, Cecilia Muñoz-Calleja, Francisco Sanchez-Madrid, Arantzazu Alfranca

**Affiliations:** ^1^ Immunology Department, Hospital Universitario de La Princesa, Instituto de Investigación Sanitaria Princesa (IIS-IP), Madrid, Spain; ^2^ Department of Immunology and Oncology, Centro Nacional de Biotecnologia/Consejo Superior de Investigaciones Científicas (CSIC), Madrid, Spain; ^3^ Department of Macromolecular Structures, Centro Nacional de Biotecnologia/Consejo Superior de Investigaciones Científicas (CSIC), Madrid, Spain; ^4^ Immunogenetics and Histocompatibility, Instituto de Investigación Sanitaria Puerta de Hierro-Segovia de Arana, Madrid, Spain; ^5^ Department of Medicine, School of Medicine, Universidad Autónoma of Madrid, Madrid, Spain; ^6^ Cardiovascular Centre for Biomedical Research Network (CIBER CV) Health Institute Carlos III, Madrid, Spain

**Keywords:** T cell, adaptive immunity, COVID-19, vaccination, immune profile

## Abstract

The immune response promoted by SARS-CoV-2 vaccination is relevant to develop novel vaccines and optimized prevention strategies. We analyzed the adaptive immunity in healthy donors (HD) and convalescent individuals (CD), before and after administering BNT162b2 vaccine. Our results revealed specific changes in CD4+ T cell reactivity profile in vaccinated HD and CD, with an increase in S1 and S2 positive individuals, proportionally higher for S2. On the contrary, NCAP reactivity observed in HD and CD patients was no longer detectable after vaccination. Despite the substantial antibody response in CD, MPro-derived peptides did not elicit CD4+ lymphocyte activation in our assay in either condition. HD presented an increment in anti-S and anti-RBD IgG after first dose vaccination, which increased after the second vaccination. Conversely, anti-S and anti-RBD IgG and IgA titers increased in already positive CD after first dose administration, remaining stable after second dose inoculation. Interestingly, we found a strong significant correlation between S1-induced CD4+ response and anti-S IgA pre-vaccination, which was lost after vaccine administration.

## Introduction

The development of humoral and cellular immunity against SARS-CoV-2, the causative agent of new coronavirus disease (COVID-19) (1), has been the subject of numerous studies, given its importance in the pathogenesis of the disease and its usefulness from a diagnostic and epidemiological perspective (2, 3). SARS-CoV-2-reactive T lymphocytes predominantly recognize peptides belonging to spike (S) protein, followed by membrane protein (VME1) and nucleoprotein (NCAP), although reactivity against other viral proteins has been described. Phenotypic characterization of T lymphocytes indicates they are mostly CD4+ Th1 (3, 4), with characteristics of effector (T_EM_) or central (T_CM_) memory cells (5, 6). IgG against S protein was found in 90% of patients 6-8 months after infection (7, 8). Similarly, specific CD4+ and CD8+ cells were found respectively in 89% and 50-74% of patients 6-9 months after infection (9).

Numerous studies show the presence of T lymphocytes reactive against SARS-CoV-2 epitopes in a variable percentage of healthy individuals (3, 4, 10–13). In most cases, these epitopes have a high degree of homology with sequences present in common cold coronaviruses. These lymphocytes could be responsible for heterologous immunity, conferring resistance to infection by SARS-CoV-2 or leading to milder COVID-19 symptoms, but no studies confirm this hypothesis.

Officially approved COVID-19 mRNA vaccines include BNT162b2 and mRNA-1273, which require two doses administered 3-4 weeks apart. Recent reports describe kinetics of humoral and cellular responses after vaccine administration (14–17); however, there are no detailed analysis of immune profile changes in patients before and after vaccine administration.

In the present study, we conducted a comprehensive characterization of CD4+T and B cell response in healthy donors (HD) and convalescents (CD) pre-and post-vaccination. We provide evidence for specific changes in T cell reactivity profile, which include increased S1 and mainly S2 specific CD4+ lymphocytes, and a loss of reactivity against NCAP in vaccinated subjects. Likewise, we detected an increment in anti-S and anti-RBD antibodies that peaked after first dose administration in CD. Interestingly, we did not detect MPro-elicited CD4+ activation despite the overt humoral response against this protein in CD. Conversely, we found a strong significant correlation between S1-induced CD4+ response and anti-S IgA in CD, which was lost after vaccination.

## Materials And Methods

### Study Population

We recruited 45 individuals, 23 of which had a history of COVID-19 confirmed by PCR, and were enrolled during the first of the pandemic in Spain. The remaining 22 HD, negative for SARS-CoV-2-specific PCR and/or serologic analyses, were recruited from the onset of the pandemic until December 2020. The study population included mostly healthcare workers from the Hospital Universitario de La Princesa, Madrid (33), related contacts (10), and hospitalized patients (2): **Supplementary Table 1** shows the demographic and clinical characteristics of this population. We analyzed T cell immunity in 21 patients, including HD (n=10), and CD (n=11) diagnosed for COVID-19 during the first pandemic wave after testing positive for SARS-CoV-2 by qPCR, serology or both. Both HD and CD were administered BNT162b2 vaccine (BioNTech, Pfizer). After administering a second dose of the vaccine, a minimum of 5-7 week period was required before performing T cell stimulation assays. Pre-vaccine determination of antibody titer in CD was done at 3, 6 and 10 months after infection. Likewise, we measured antibody level in these patients 15-20 days after first vaccination and 5-7 weeks after the second vaccination. Similarly, antibody titer in HD was analyzed in parallel to last pre-vaccine (10 months) and both post-vaccine determinations in CD.

Methods for PBMC Isolation and Culture, Flow Cytometry, Expression of SARS-CoV-2 proteins, ELISA for detection of SARS-CoV-2 antibodies, HLA typing, and Statistical Analysis are detailed in the **Supplementary Material**.

## Results

### Analysis of T Cell Activity in Response to SARS-CoV-2 Specific Peptides

To analyze changes in immune specificity profile after administering BNT162b2 vaccine, we recruited 23 CD and 22 HD (**Supplementary Table 1**). We evaluated T cell response after stimulation with peptide pools from SARS-CoV-2 proteins (S1, S2, RBD domain, VME1, NCAP and Mpro) in 10 HD and 11 CD. In addition, we determined the presence of antibodies against S protein, RBD domain, NCAP and Mpro in the entire study population.

T cell activity was determined by an activation induced marker (AIM) assay, based on detecting surface expression of CD25 and CD69 in CD4+ lymphocytes by flow cytometry (**Supplementary Figure 1**). To compare results obtained with our approach with previously published AIM assays (3), we analyzed parallel expression of OX40/CD137 in activated CD4+ lymphocytes of a group of 7 exposed donors. In addition, we determined IFNγ expression to evaluate functional activity in these cells (**Supplementary Figure 2**). In basal conditions, the percentage of CD69+CD25+ cells was significantly higher than that of OX40+CD137+ (p<0.05), although similar to the proportion of IFNγ+ CD4+ lymphocytes. After Staphylococcal enterotoxin B (SEB) stimulation, we observed a substantial increase in the specific percentage of CD69+CD25+ lymphocytes, being significantly higher than the percentage of IFNγ+ and OX40+CD137+ found in SEB-activated cells (p<0.001) (**Supplementary Figures 2A, C**). We then determined the proportion of patients positive for each individual labeling and for staining combinations in SEB- and peptide-activated CD4+ lymphocytes. In SEB-treated cells, we found that all patients were positive for activation with CD25/CD69 together with one or two additional markers. In peptide-activated lymphocytes, we found a positive staining with at least one labeling in the presence of either S1 or S2 in 11/14 assays. CD25/CD69 activation was also detected with one or two additional approaches in most cases (9/11 positive cultures). Of note, 4/11 peptide-activated cultures did not show OX40/CD137 staining while co-expressing both CD25/CD69 and IFNγ (**Supplementary Figure 2B**). In view of these results, we considered CD25/CD69 labeling as a sensitive approach suitable to analyze CD4+ T cell response to SARS-CoV-2.

### Immune Specificity Profile in Healthy and Convalescent Subjects

**Figure 1:**
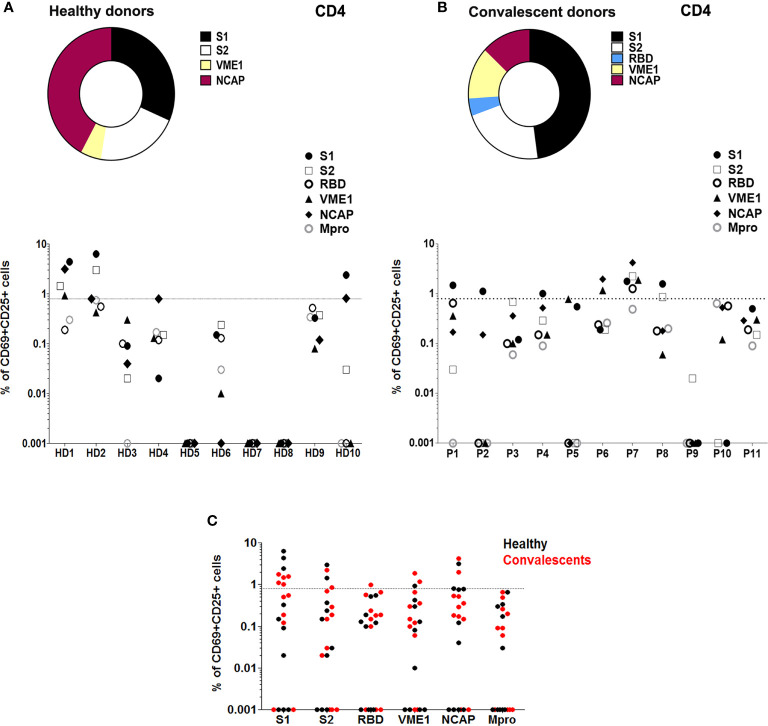
T cell reactivity profile to SARS-CoV-2 in healthy and convalescent individuals before vaccination. **(A)** *Upper panel*, proportion of HD (n=10) with SARS-Cov-2-reactive CD4+ T cells after stimulation with different peptide pools as indicated. *Lower panel*, T cell activity against different peptide pools measured in each HD. **(B)** *Upper panel*, proportion of CD (P; n=11) with SARS-Cov-2-reactive CD4+ T cells after stimulation with different peptide pools as indicated. *Lower panel*, T cell activity against different peptide pools measured in each CD. **(C)** T cell activation in HD (black) and CD (red), in the presence of specific SARS-CoV-2 peptide pools. T cell activity was measured as specific percentage, considered positive when ≥ 0.85% (dotted lines).

The analysis of SARS-CoV-2-induced T cell activation pre-vaccination showed that 40% HD displayed activation of CD4+T cells in the presence of peptides from S1 (30%), S2 (20%), NCAP (40%), and VME1 (10%) (**Figures 1A, C**). Assessment of T cell activation in CD also revealed specific responses to SARS-CoV-2 in CD4+ (54%), which were triggered by peptides from S1 (45%), S2 (18%), RBD (9%), NCAP (18%) and VME1 (18%) (**Figures 1B, C**). Similar results were obtained when stimulation index (SI) was used to estimate specific T cell activity (**Supplementary Figures 3A, B**).

**Figure 2:**
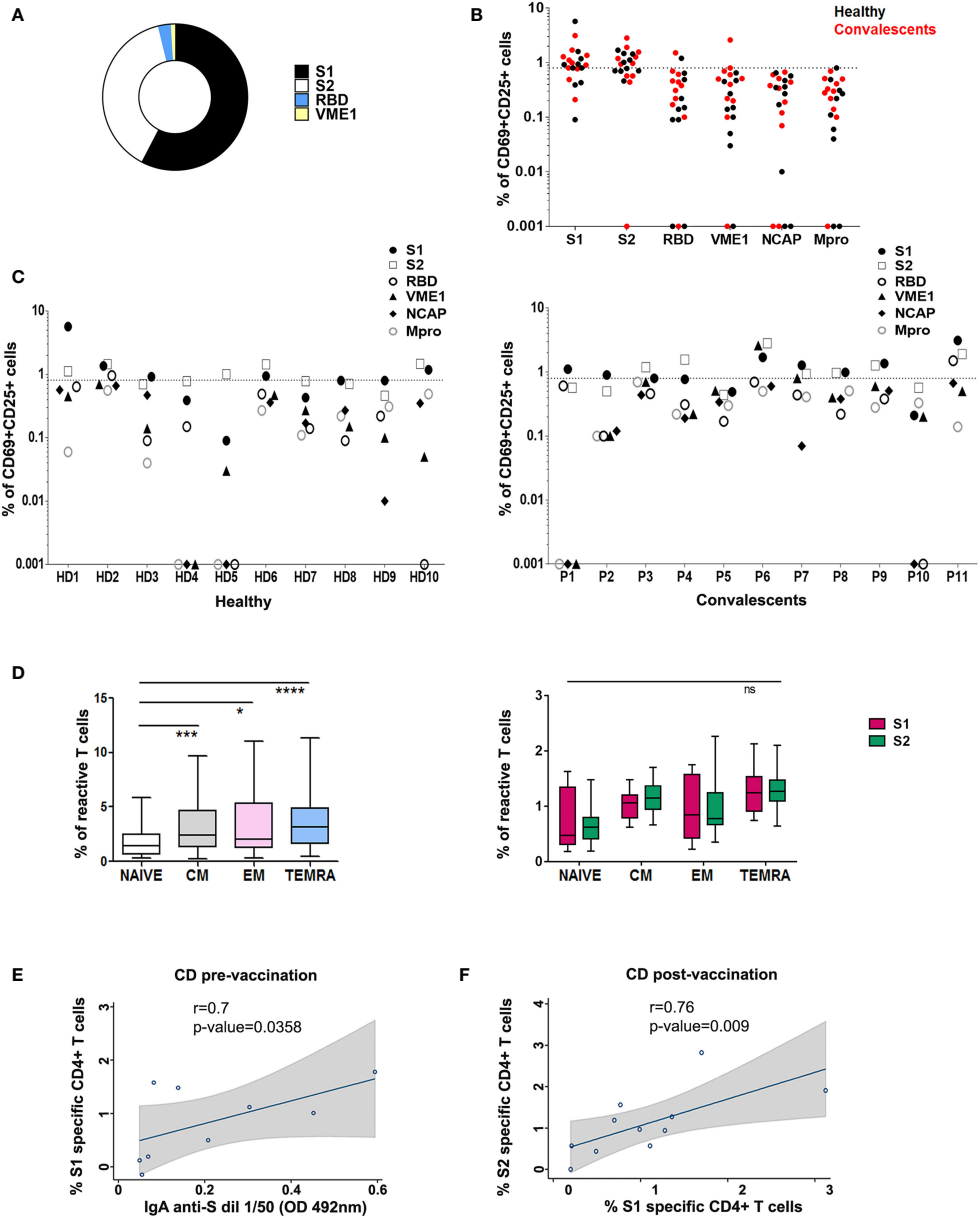
T cell reactivity profile to SARS-CoV-2 in healthy and convalescent individuals after vaccination. **(A)** Proportion of HD and CD (P) (n=21) with SARS-CoV-2 reactive CD4+ T cells after stimulation with different peptide pools as indicated. **(B)** Post-vaccination CD4+ T cell activation in HD (black) and CD (red), in the presence of specific peptide pools. **(C)** CD4+ activity against different peptide pools in each HD (left) and CD (P; right). T cell activity was measured as specific percentage, considered positive when ≥ 0.85% (dotted line). **(D)** *Left*, CD4+ T memory subsets specific for SARS-CoV-2 peptide pools in vaccinated donors. *p < 0.05; ***p < 0.001; ****p < 0.0001; *Right*, Comparison of CD4+ T memory subsets specific for S1 and S2 peptide pools in vaccinated donors; ns, non-significant. **(E)** Correlation between SARS-CoV-2 S1 specific CD4+ T cells (%) and IgA anti-S in CD pre-vaccination. **(F)** Correlation between SARS-CoV-2 S1 specific CD4+ T cells and SARS-CoV-2 S2 specific CD4+ T cells (%) in CD post-vaccination. Spearman correlations were calculated. Fitted linear prediction and its 95% confidence interval (transparent gray shadow) are shown.

Despite T cell immunity in several HD, we could not detect antibodies against SARS-CoV-2 S protein, RBD domain, Mpro or NCAP of either IgG, IgA or IgM isotypes in HD (**Supplementary Figure 4A**). Conversely, the analysis of humoral response in CD 10 months after infection revealed detectable levels of IgG against-S, RBD, NCAP and Mpro proteins, and low to undetectable concentrations of IgA and IgM antibodies against any of these targets (**Supplementary Figure 4B**). Interestingly, we observed a strong significant correlation between S1-induced CD4+ response and anti-S IgA in CD (r=0.7; p=0.0358; **Figure 2E**).

### T Cell Specificity Profile in Vaccinated Donors

**Figure 3:**
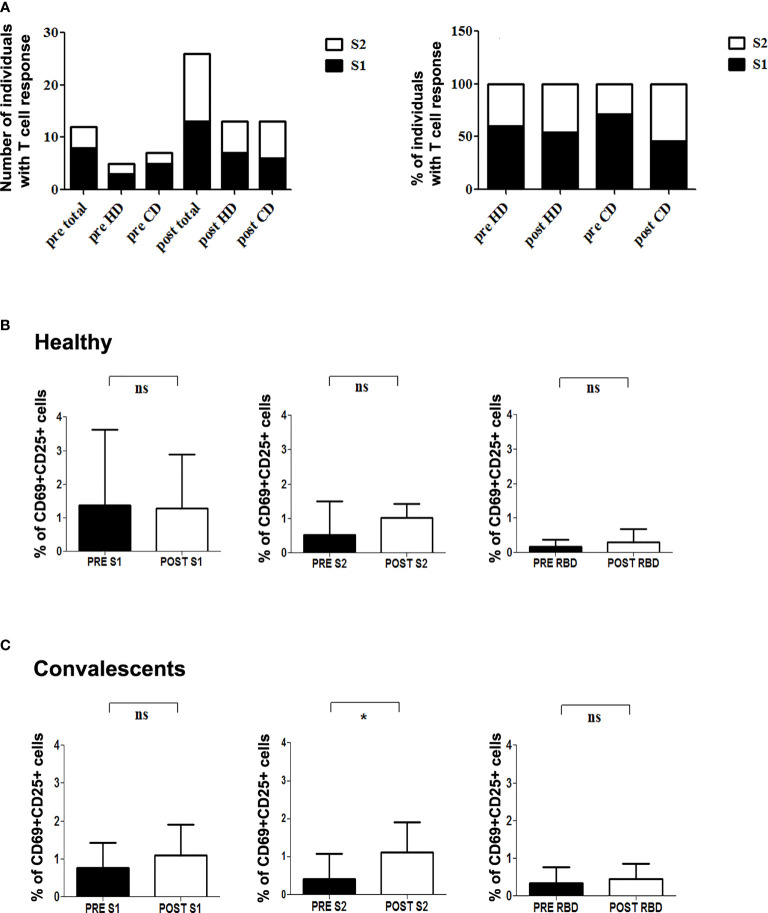
T cell reactivity to SARS-CoV-2 S protein in healthy and convalescent individuals before and after vaccination. **(A)** *Left*, absolute number of individuals with CD4+ reactive lymphocytes against S1 (black) and S2 (white) before (pre) and after (post) vaccination. *Right*, Relative percentage of S-protein positive donors reactive for either S1 (black) or S2 (white) in each condition. **(B, C)** Graphics show specific percentage of HD **(B)** and CD **(C)** before (pre) and after (post) vaccination in the presence of S1 (left), S2 (middle) and RBD (right) peptide pools. Statistical analysis was performed with the paired Student’s t-test. *p=0.045; ns, not significant.

We next assessed modifications in individual immune specificity profiles of HD and CD 5-7 weeks after vaccination, and found that most patients showed specific CD4+ activation (60% HD and 81% CD). Predominant CD4+ responses were directed against peptides from S1 (66%), S2 (57%), RBD (9%), and VME1 (9%) (**Figures 2A, B**). HD showing S1- or S2-induced CD4+ activation in basal conditions maintained these specificities, whereas CD4+ reactivity against NCAP and VME1 peptides was not detectable after vaccination (**Figures 1A** and **2C**). Similarly, basal activation observed in CD in the presence of peptides from S1, S2 and VME1 remained detectable after vaccine administration, whereas NCAP-induced responses were not (**Figures 1B** and **2C**). SI yielded results comparable to specific percentage also in vaccinated donors (**Supplementary Figure 3C**). Of note, the percentage of CD4+ responsive to S1 and S2 peptides strongly correlated in vaccinated CD (r=0.79; p=0.009; **Figure 2F**), but not pre-vaccination (**Supplementary Figure 3E**). After vaccination an increase in S1 and S2 positive patients was observed in both HD and CD groups, which was proportionally higher for S2 (**Figure 3A**). Furthermore, we found a significant increase in the extent of S2-specific CD4+ response in CD (p<0.05), which was not detected in HD (**Figures 3B, C**).

SARS-CoV-2 mRNA vaccination promotes memory CD4+ T cell responses (18). We, therefore, sought to characterize SARS-CoV-2-specific memory CD4+ T cell subpopulations in vaccinated donors included in our study. We detected specific CD4+ T_CM_ (CD45RO+ CD27+), T_EM_ (CD45RO+ CD27-) and mainly terminally differentiated effector (T_EMRA_; CD45RO- CD27-) memory subsets in vaccinated individuals (**Figure 2D**, *left*). When analyzing memory subsets in HD and CD separately, we found that CD but not HD specifically showed an increase in T_EM_ (**Supplementary Figure 3D**). Of note, we did not observe significant differences between S1- and S2-specific memory CD4+ T lymphocytes (**Figure 2D**, *right*). Finally, SARS-CoV-2 mRNA vaccine has been reported to elicit a transient increase in specific circulating T follicular helper clls (cTfh) (18). In addition, PD-1hi cTfh are thought to represent a subset of recently activated cTfh cells (7). We have analyzed the percentage of SARS-CoV-2-specific cTfh and PD-1hi cTfh in healthy and convalescent vaccinated donors, finding non-significant differences in either S1 or S2-responsive cTfh between both groups (**Supplementary Figure 5**).

### Antibody Specificity Profile in Vaccinated Donors

**Figure 4:**
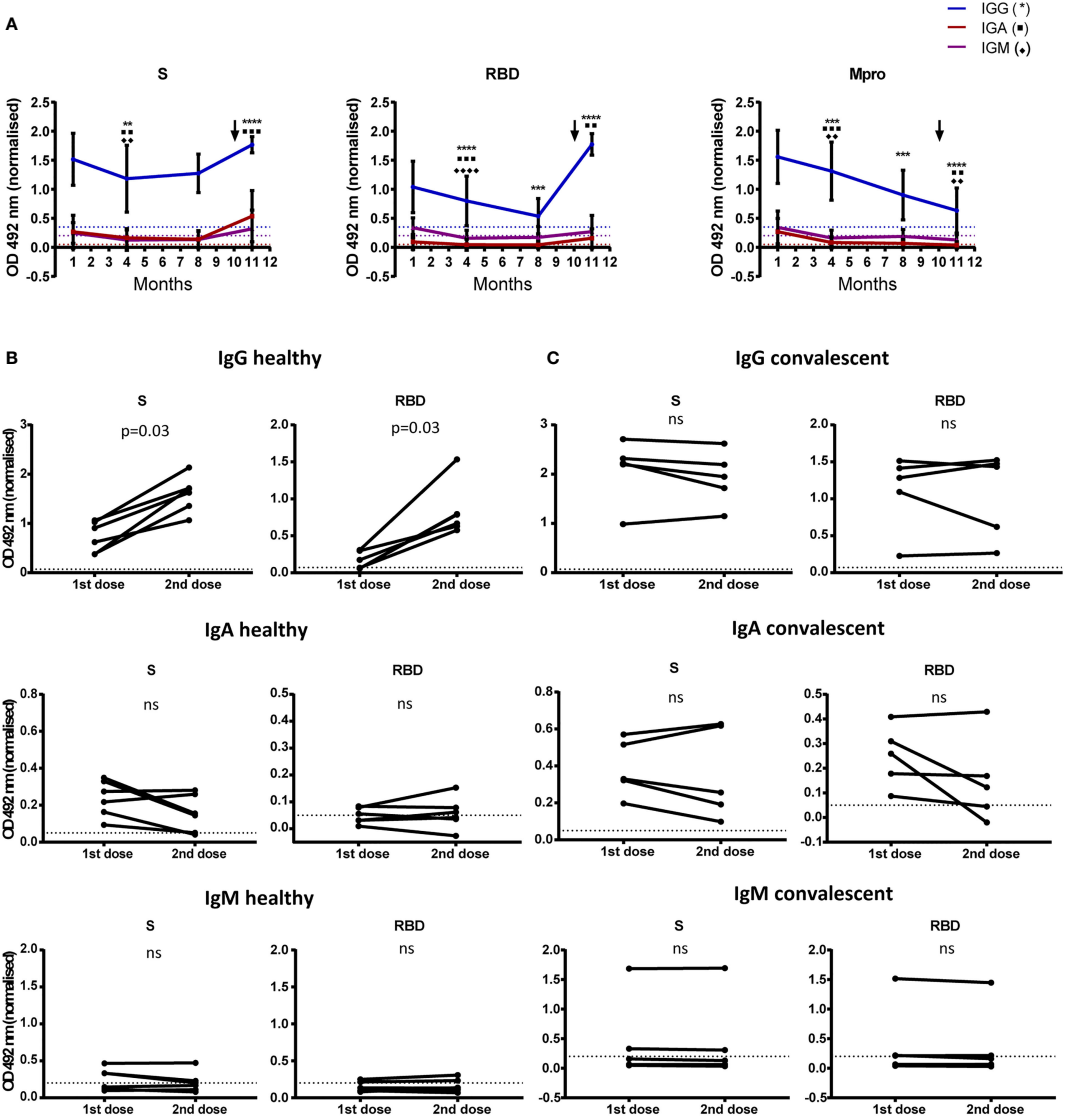
Antibody levels to SARS-CoV-2 before and after vaccination. **(A)** 11 month-follow up of 18 CD. The mean and SD of optical density at 492 nm are shown for IgG, IgA and IgM. Dilutions of 1/200 for IgG and 1/50 for IgA and IgM were used. Antibody detection at month 1 corresponds to samples collected 3 months after disease onset. First dose of the vaccine was administered at month 9 (month 11 after disease onset; arrow). Determination of antibodies at month 11 corresponds to second vaccine dose administration. Optical density was normalized using the signal obtained with a pool of positive sera.**p < 0.01; ***p < 0.001; ****p < 0.0001. **(B, C)** Comparison of SARS-CoV-2 antibody levels determined after each vaccine dose. Paired graphs show anti-S and RBD IgG, IgA and IgM in sera of 5 HD **(B)** and 5 CD **(C)** Sera dilutions of 1/3200 for IgG and 1/50 for IgA and IgM were used. Optical density at 492 nm was normalized using the signal obtained with a pool of positive sera. Statistical significance was analyzed by Wilcoxon test. Positivity threshold was calculated as 3 SD above mean in healthy donors in all cases (dotted lines). ns, non significant.

We determined vaccine-induced changes in humoral response in HD and CD, 15-20 days after first dose and 5-7 weeks after the second dose. Serial antibody determination in CD at months 3, 6 and 10 after infection showed that, while anti-S IgG level remained rather stable over time, anti-RBD IgG and anti-Mpro IgG experienced a constant decrease. Likewise, titers of specific IgA and IgM decreased from month 3 to reach low (anti-S IgA) to undetectable levels at month 6 after disease onset in all cases (**Figure 4A**). Furthermore, levels of anti-S and anti-RBD IgG and IgA significantly increased in CD after first dose inoculation (**Figure 4A**), reaching levels with no further increment after completing the vaccination schedule (**Figure 4C**). In HD, we observed an increment in anti-S and anti-RBD IgG after first dose inoculation, which was further increased after the second injection (**Figure 4B**). Levels of anti-S and anti-RBD IgA and IgM also rose after the first dose in HD: 100% anti-S IgA; 60% anti-RBD IgA; 40% anti-S IgM; and 40% anti-RBD IgM. Nonetheless, we did not detect any additional increase in these isotypes after the second vaccination (**Figure 4B**).

## Discussion

In the present study, we sought to investigate changes in immune specificity profile after vaccination against SARS-CoV-2. We first analyzed pre-vaccine specific immune adaptive responses, and identified reactive T lymphocytes in both HD and CD. We selected NCAP and S proteins because they contain peptides that elicit CD4+ T cell response in a great proportion of HD and CD (4, 11). In fact, several HLA-DRB1-restricted immunodominant peptides have been described for both proteins (4). Conversely, VME1 protein has been reported to contain HLA-DRB1-restricted immunodominant peptides and drive CD4+ activation only in CD (4). However, other authors detected overt VME1-induced CD4+ reactivity also in HD (3, 19). We also included peptides from Mpro, a non-structural protein contained in Orf1, because of its high homology with the main protease of SARS-CoV and the strong humoral response evoked by this protein in CD (20). The heterogeneous specificity observed in CD4+ responses in both HD and CD (**Figures 1**, **2**) could be due to differences in class II HLA profile (4). However, high-resolution analysis of HLA-DRB1 did not show a clear relationship between donor genotype and CD4+ activation profile (**Supplementary Table 2**).

The detection of SARS-CoV-2-specific T cells in HD has been attributed to cross-reaction with peptides from common cold coronaviruses (3, 5, 12, 21), and cross-reactive CD4+ T cell receptors for S protein peptides have been recently characterized (22). HD recruited for our study were either healthcare workers or related contacts, so exposure to SARS-CoV-2 cannot be completely ruled out. In fact, we detected T cell response against NCAP in a percentage of HD (**Figures 1A, C**), which has been connected by several authors with exposure to SARS-CoV-2. In this regard, T cell activation induced by NCAP was detected both in exposed individuals and in HD recruited in 2020, but not in HD recruited in 2019 (10). Likewise, NCAP-specific T cells were identified only in exposed subjects and CD, but not in prepandemic or unexposed individuals (23). In accordance with our data, a CD4+ specificity profile that includes T cell reactivity against S, VME1 and NCAP peptides has been reported in CD (3, 19).

A recent paper shows that the percentage of anti-S specific CD4+ peaks 7 days after BNT162b2 first-dose administration in CD, decreasing 7 days later and not recovering with a second dose. Conversely, HD showed a significant increase in S-specific CD4+ cells after completing the vaccination schedule, reaching the same level of response as CD (14). However, this study did not analyze the CD4+ response specifically against S1 and S2. Interestingly, we have found a strong correlation between S1- and S2-specific CD4+ lymphocytes in vaccinated CD, which show a relatively higher response for S2 in both HD and CD (**Figure 3A**). Thus, a qualitative change in T cell response against S protein takes place in vaccinated donors; nevertheless, further studies are required to establish the actual significance of these findings in terms of protection against SARS-COV-2 infection. On the other hand, NCAP-induced T cell activation observed in some patients is no longer detectable after vaccination (**Figures 1A, 2A–C** and **Supplementary Figure 3**). It is conceivable that vaccine-induced expansion of S-specific CD4+ clones dilutes the relative frequency of NCAP-activated T lymphocytes, hampering their detection in our short-term *in vitro* activation assay. In addition, T cells specific for NCAP epitope Nuc322-331 in CD undergo a gradual decrease in their activation state, together with an increment in lymph node homing and proliferation potential, which could influence their identification in short-term activation-based assays (24).

In addition to mRNA vaccines, SARS-CoV-2 vaccines based on adenoviral vectors (ChAdOx1 nCoV-19, Ad26.COV2.S, Ad5-nCoV, etc), synthetic S protein (NVX-CoV2373), and whole-cell inactivated vectors (BBV152) were found to elicit a robust CD4+ T cell response after first and/or second dose, mainly composed of IFNγ− and TNFα-producing TH_1_ cells (25). However, there is no detailed characterization of T cell activation profile in these patients.

Our data on humoral response in CD, are also in line with previous reports showing constant levels of anti-S IgG up to 6 months after infection, together with progressive decrease of anti-RBD and anti-NCAP IgG (7). Despite the strong antibody response against Mpro in CD, we did not detect Mpro-induced T cell activation in these subjects (**Figure 1B**). A possible explanation is that Mpro protein processing does not yield peptides immunogenic enough to elicit a T cell response detectable in our assay. It is also possible that peptide pool composition does not adequately reflect actual *in vivo* Mpro protein processing.

Following our results, some authors have recently reported a maximum increase of anti-S and anti-RBD IgG with a single dose of BNT162b2 vaccine in CD (14–17). Nevertheless, in contrast to our data, an increase of anti-S IgM and IgA levels upon first dose administration was described in HD, which was further enhanced 1 week after the second dose (14). However, we determined antibody titers 5-7 weeks after complete vaccination, thus we cannot rule out that IgM and IgA levels had already decreased at this time point. As expected, anti-Mpro antibodies did not increase after vaccination, whereas at the end of the study, IgG, and to a lesser extent IgA, were readily detectable with significantly higher levels than in HD (**Figure 4A** and **Supplementary Figure 4B**). The presence of detectable titers of anti-Mpro IgG in CD but not in HD may provide a useful tool to reliably discriminate between vaccinated healthy population and COVID-19 patients.

## Data Availability Statement

The original contributions presented in the study are included in the article/**Supplementary Material**. Further inquiries can be directed to the corresponding author.

## Ethics Statement

This study was approved by the local Research Ethics Committee (CEIm Hospital Universitario de La Princesa; register number 4070), and it was carried out following the ethical principles established in the Declaration of Helsinki. The patients or their representatives provided their written informed consent to participate in this study.

## Author Contributions

AA, FS-M, CM-C, and EM-G contributed to research design and data analysis, and either wrote or revised the manuscript. LE-P and PM-F contributed to data acquisition and analysis. NR and PV-T contributed to acquisition of the data. CV performed HLA genotyping and revised the manuscript. HR and JC generated recombinant proteins and revised the manuscript. MV-G and JR produced ELISA plates and revised the manuscript. All authors contributed to the article and approved the submitted version.

## Funding

This work was supported by grants to AA: FIS PI19/01491 (Fondo de Investigación Sanitaria del Instituto de Salud Carlos III with co-funding from the Fondo Europeo de Desarrollo Regional FEDER) and Sociedad Cooperativa de Viviendas Buen Suceso, S.Coop.Mad. To AA and FS-M: CIBER Cardiovascular from the Instituto de Salud Carlos III (Fondo de Investigación Sanitaria del Instituto de Salud Carlos III with co-funding from the Fondo Europeo de Desarrollo Regional; FEDER). To FS-M: SAF2017-82886-R (Spanish Ministry of Economy and Competitiveness MINECO)), HR17-00016 (“La Caixa” Banking Foundation), “Fondos Supera COVID19” (Banco de Santander and CRUE), “Ayuda Covid 2019” and “Inmunovacter” REACT-UE (Comunidad de Madrid). To MV: Spanish National Research Council (CSIC, project number 202020E079 and CSIC-COVID19-028).

## Conflict of Interest

The authors declare that the research was conducted in the absence of any commercial or financial relationships that could be construed as a potential conflict of interest.

## Publisher’s Note

All claims expressed in this article are solely those of the authors and do not necessarily represent those of their affiliated organizations, or those of the publisher, the editors and the reviewers. Any product that may be evaluated in this article, or claim that may be made by its manufacturer, is not guaranteed or endorsed by the publisher.
