# Supplementary figures and images for: CD4+ T Cell Immune Specificity Changes After Vaccination in Healthy And COVID-19 Convalescent Subjects
Source: Front Immunol. 2022 Jan 19;12:755891. doi: 10.3389/fimmu.2021.755891 (PMC8807633; doi:10.3389/fimmu.2021.755891)

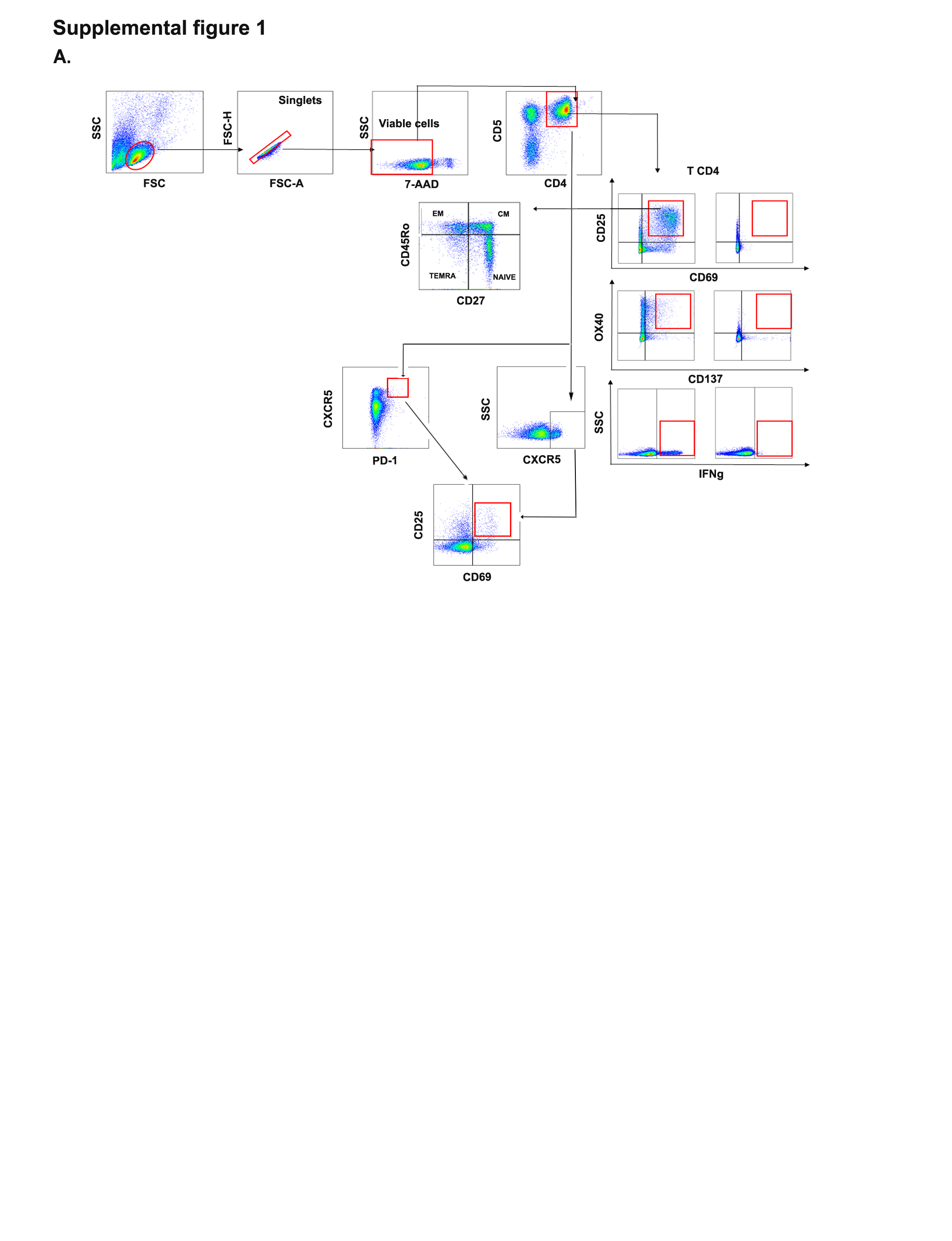

Supplement: Supplementary file 1 [file Image_1.tif]

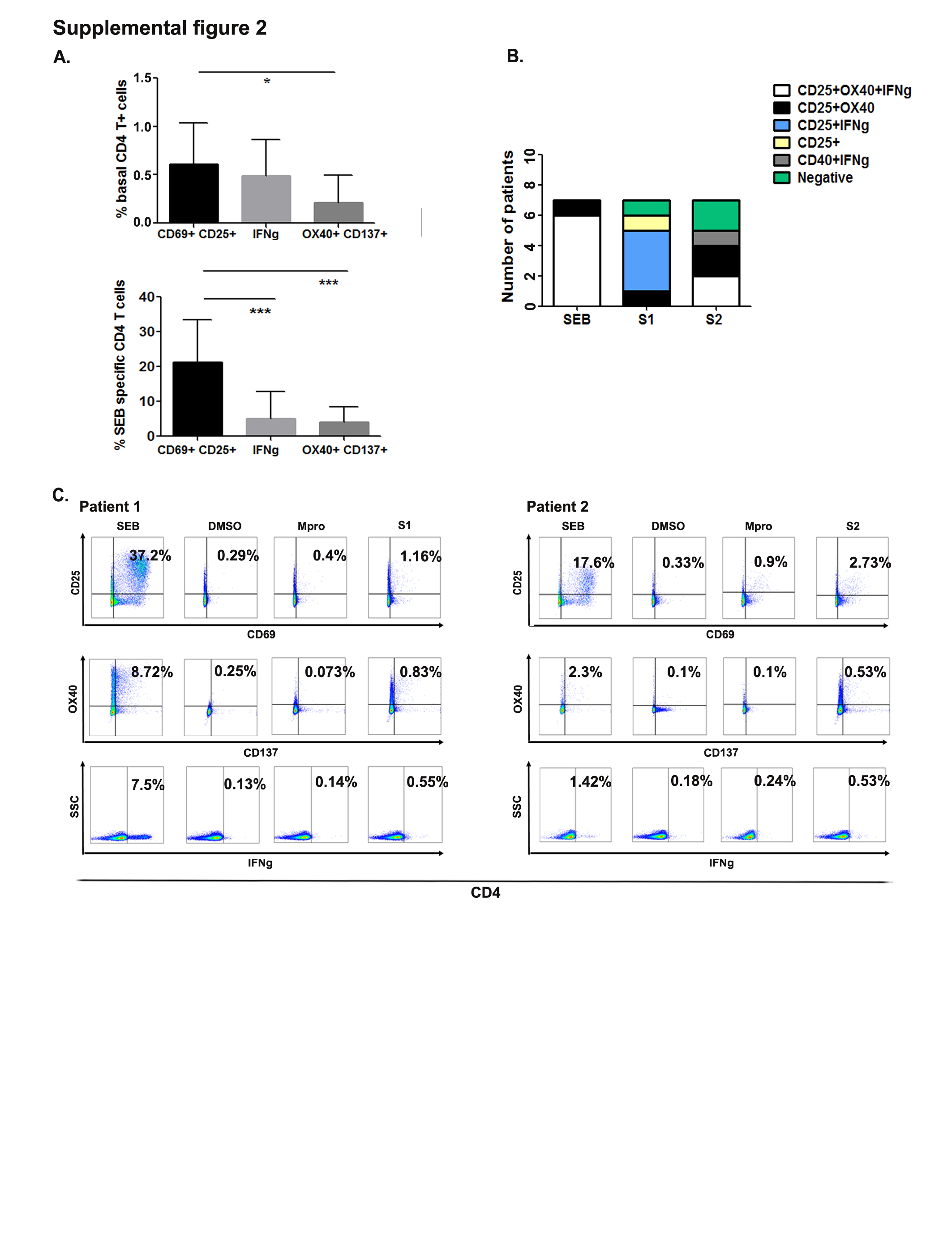

Supplement: Supplementary file 2 [file Image_2.tif]

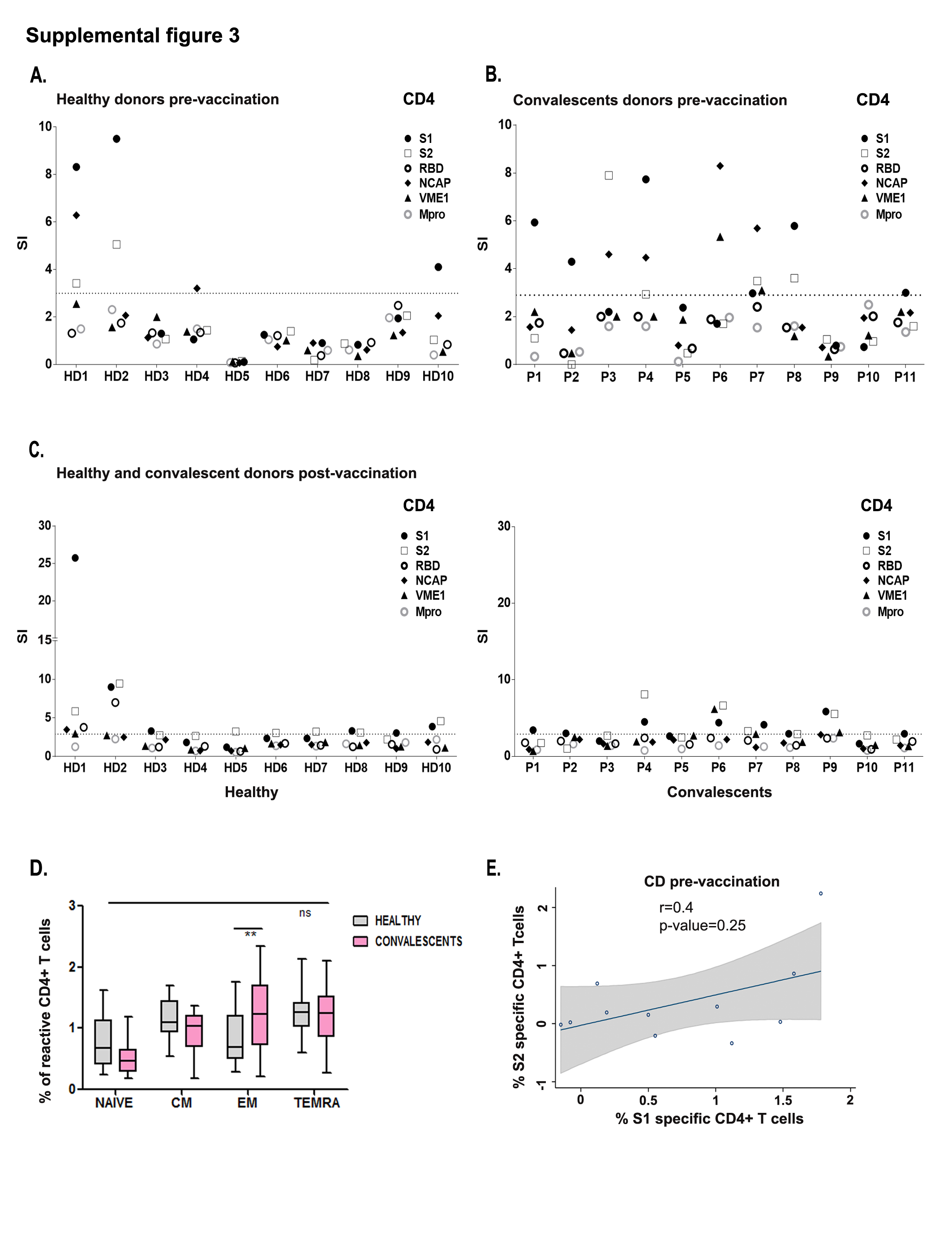

Supplement: Supplementary file 3 [file Image_3.tif]

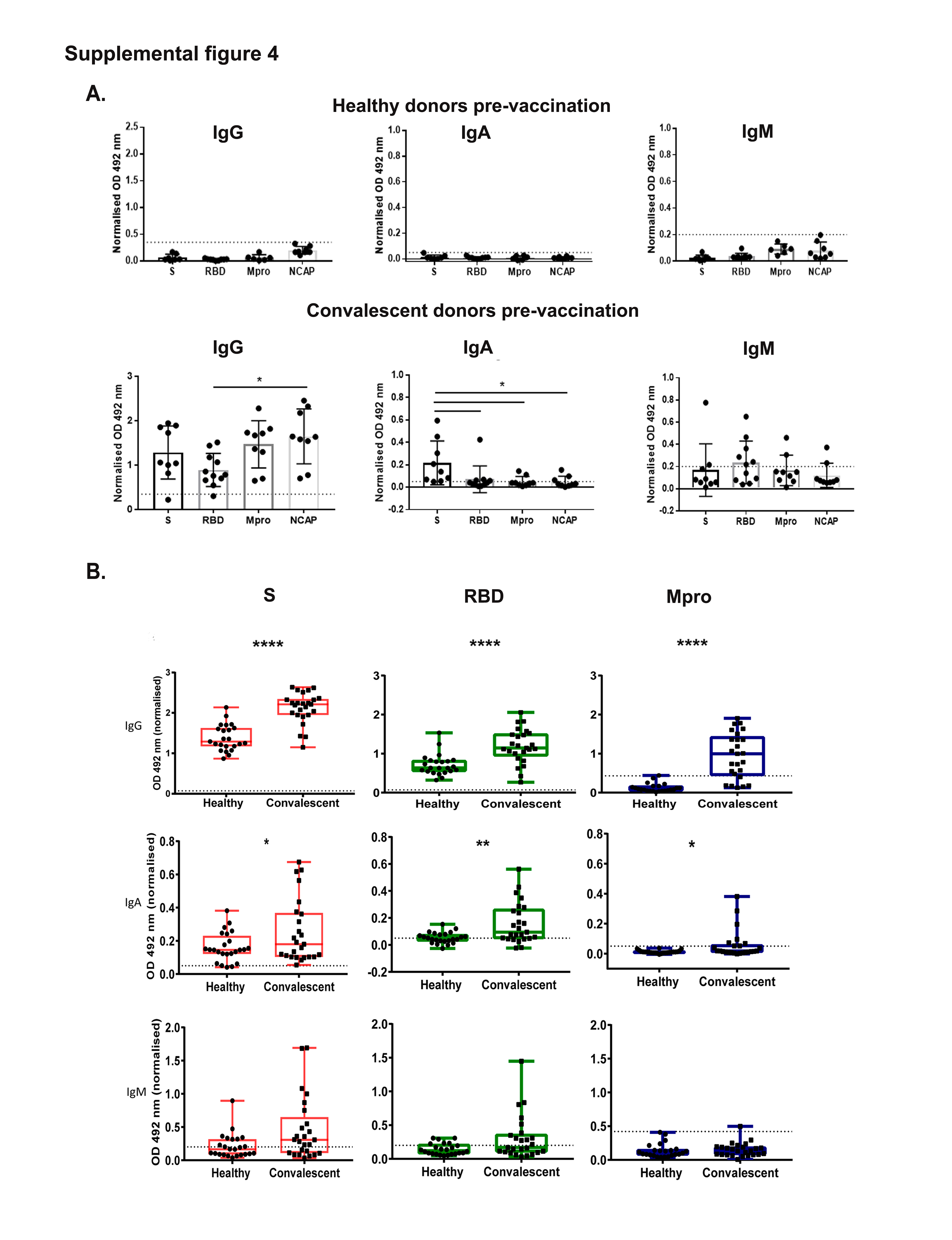

Supplement: Supplementary file 4 [file Image_4.tif]

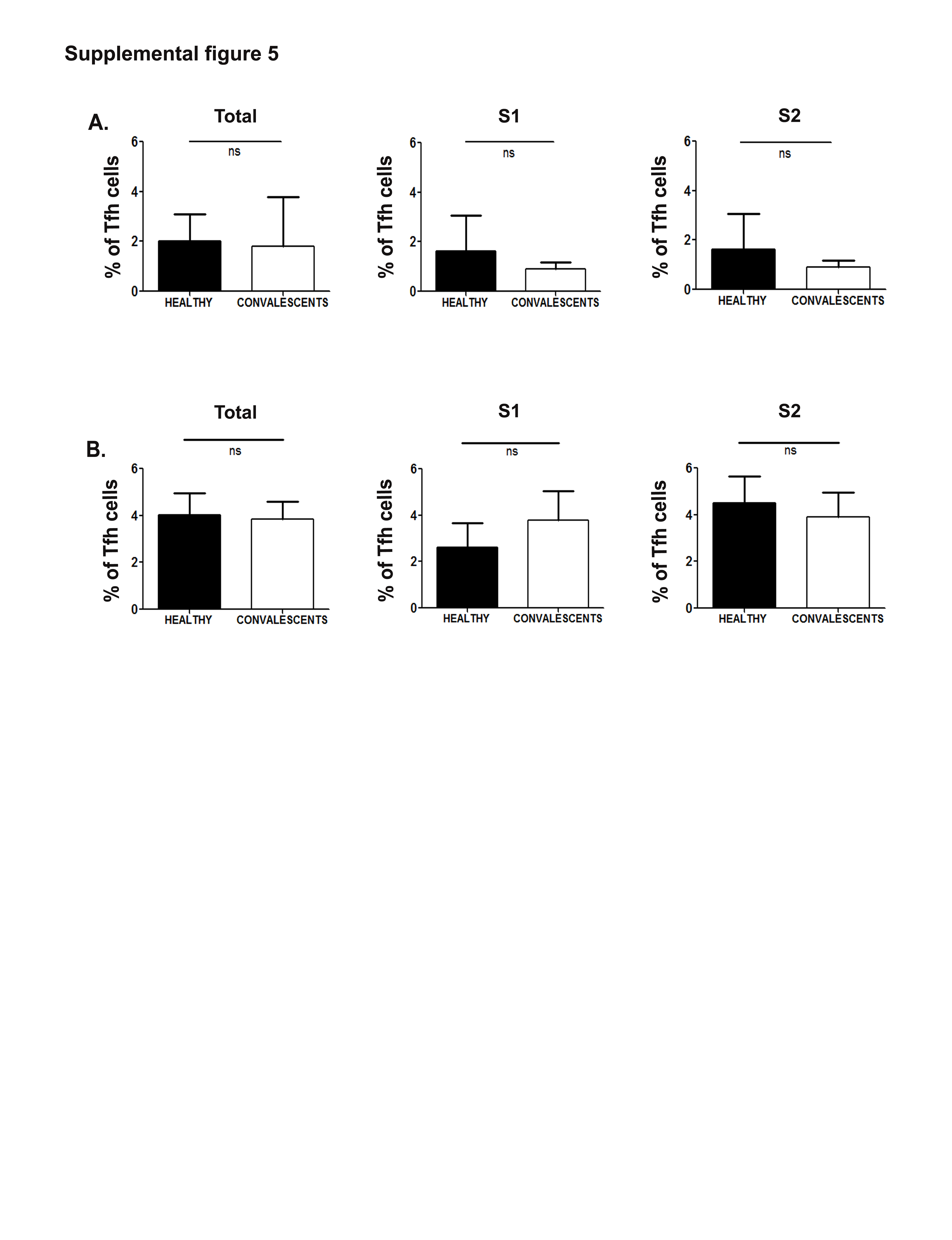

Supplement: Supplementary file 5 [file Image_5.tif]
