# Supplementary material for: CD4+ T Cell Immune Specificity Changes After Vaccination in Healthy And COVID-19 Convalescent Subjects
Source: Front Immunol. 2022 Jan 19;12:755891. doi: 10.3389/fimmu.2021.755891 (PMC8807633; doi:10.3389/fimmu.2021.755891)
Supplement: Supplementary file 6 [file DataSheet_1.docx]

Supplementary Material

**SUPPLEMENTARY MATERIALS AND METHODS**

**PBMC Isolation and Culture**

For T cell activation assays, 10 ml of whole blood were collected in EDTA tubes. Peripheral blood mononuclear cells (PBMCs) were isolated by density-gradient sedimentation using Ficoll-Paque (Pan-Biotech), following manufacturer’s instructions. Isolated PBMCs were cryopreserved in fetal bovine serum (HyClone TM) containing 10% DMSO (Inilab) and subsequently maintained at -80ºC, or liquid N2 for long-term storage. For flow cytometry assays, PBMCs were thawed using 10ml of medium RMPI 1640 (GIBCO) supplemented with 10% fetal bovine serum and 5% penicillin-streptomycin (Biowest). Then, 2x10^5^ cells per well were cultured for either 16 hours (for CD69/CD25 staining) or 24h (for OX40/CD137 and IFNγ staining) at 37ºC and 5%CO2 atmosphere, in the presence of six different SARS-COV-2 peptide pools (PepMixTM; JPT Peptide Technologies). Pools included peptides from S1, S2 and RBD from spike (S) protein, VME1 (membrane protein), NCAP (nucleoprotein) and Mpro (Cys-like protease, nsp5) (1μg/ml). For intracellular IFNγ staining, cells were added 10μg/ml Brefeldin A (Sigma Aldrich) two hours after peptide addition. A positive control was performed by stimulating cells in the presence of the staphylococcal enterotoxin B (SEB; Sigma Aldrich) (1μg/ml).

**Flow Cytometry**

After stimulation, plates were centrifuged for 6 min at 1600 rpm, and cell pellets were resuspended in 200μl PBS 1X (Lonza) containing 0.5% (w/v) BSA (Roche). Then, cells were incubated with a combination of four monoclonal antibodies: anti-human CD69-FITC/CD25-PE/CD5-PE-Cy7 as a pan-T marker/ CD4-Pacific Blue (BD Becton Dickinson) for 30 min. 7-AAD (eBioscience) was added following manufacturer´s instructions. For comparison purposes, cells were incubated in parallel with either anti-OX40-PE/CD137-BV711 (Biolegend)/CD69-FITC/CD5-PE-Cy7/CD4-Pacific Blue, or with anti-CD5-PE-Cy7/CD4-Pacific Blue/IFNγ-BV605 after permeabilization with Cytofix-Cytoperm (BD Becton Dickinson). Subsequently, cells were washed and finally resuspended in 200μl PBS 1x. Cells incubated with DMSO were included as negative stimulation control.

All the samples were acquired on a BD FACSCanto II flow cytometer (BD Becton Dickinson). The gating strategy for stimulated T cells is provided in Suppl Fig. 1: CD4+ T cells were identified as positive for CD4 and CD5 markers. Surface CD3 expression is known to decrease with TCR activation (1), so we used CD5 pan-T marker to identify T lymphocytes in our samples. Although there is a minority CD19+ subset that may co-express membrane CD5 (2), this population was avoided by selecting CD5+CD4+ co-expressing cells. Activated cells were defined as double positive for CD69 and CD25 markers. For comparative assays, activated cells were additionally defined as double positive for OX40/CD137 or IFNγ-expressing cells. To study different memory CD4+ T cell subsets, PMBCs were also stained with CD27-APC and CD45RO- PE-Cy7 (BD Becton Dickinson). Memory subpopulations were defined as follows: T_CM:_ CD45RO+ CD27+, T_EM_: CD45RO+ CD27-, and T_EMRA_: CD45RO- CD27-; naive CD4+ T cells were defined as CD45RO- CD27+. SARS-CoV-2 specific cTfh cells were defined as CD69+CD25+ double positive cells within CD4+CXCR5+ and CD4+CXCR5+PD-1hi populations. Flow cytometry data were analyzed using FlowJo software (BD Becton Dickinson).

Specific T cell response was determined by either subtracting the percentage of double positive cells in the presence of DMSO from that obtained with SEB/peptides (specific percentage), or by dividing the percentage of activated cells after stimulation with SEB/peptides and the percentage of activated cells in the presence of DMSO (stimulation index, SI). A positive result was considered for samples with specific percentage equal or above the median two-fold standard deviation of all negative controls (0.85% for CD25/CD69, 0.62% for OX40/CD137 and 0.3% for IFNγ), or with SI≥3 for CD69/CD25 and IFNγ, and ≥2 for OX40/CD137 (3).

**Expression of SARS-CoV-2 Cys-like protease (Mpro), nucleocapsid (NCAP), Spike (S) and RBD proteins**

The Cys-like protease (Mpro) and nucleocapsid (NCAP) proteins constructs were expressed in the E. coli strain BL21 Star (DE3) pLysS (ThermoFisher), and purified as described (4). The soluble S (residues 1 to 1208) and RBD (334 to 528) proteins were produced in HEK-293F or CHO cells. The S was fused to a T4 fibritin trimerization sequence, a Flag epitope and an 8xHis-tag at its C-terminus; in addition, the furin-recognition motif (RRAR) was replaced by the GSAS sequence and the A942P, K986P and V987P substitutions were introduced in the S2 portion. The S protein was purified by Ni-NTA affinity chromatography from transfected cell supernatants and it was transferred to 25 mM Hepes-buffer and 150 mM NaCl, pH 7.5, during concentration. The RBD preparation is described elsewhere (4).

**ELISA for detection of SARS-CoV-2 antibodies**

96-well Maxisorp Nunc-Immuno plates were coated with 100 μL/well of recombinant proteins diluted in borate buffered saline (BBS) (Mpro at 0.5 μg/ml; NCAP and RBD at 1μg/ml), and incubated overnight at 4ºC. Coating solutions were then aspirated, the ELISA plates were washed three times with 200 μl of PBS 0.05% Tween 20 (PBS-T) and blocked with PBS-casein (Biorad, 1x PBS blocker) for 1 hour at room temperature. The plates were washed again with PBS-T and 100 μl of patient serum/plasma sample diluted in PBS-casein, 0.02% Tween-20, as indicated in figure legends, was added and incubated for 2 hours at room temperature. After washing, 100 μl/well of the indicated detection antibody (AffiniPure Rabbit Anti-Human IgM, Fcµ fragment specific. HRPO, AffiniPure Rabbit Anti-Human Serum IgA, α chain specific. HRPO, or AffiniPure Rabbit Anti-Human IgG, Fcγ fragment specific. HRPO, all from Jackson Labs) was added and incubated for 1 hour at room temperature. The plates were washed with PBS-T four times and incubated at room temperature in the dark with 100 μl/well of Substrate Solution (OPD, Sigma prepared according to the manufacturer’s instructions, typically for 3 minutes). 50 μl of stop solution (3M H_2_SO_4_) were then added to each well and the optical density (at 492nm) of each well was determined using a microplate reader.

**HLA typing studies**

The HLA-DRB1 genes were studied on genomic DNA samples by polymerase-chain reaction and reverse hybridization with sequence-specific oligonucleotide probes, using commercial reagents based on the xMAP® Technology (LABType SSO, One Lambda-Thermo Fisher Scientific, West Hills CA). These probes distinguish all two-digit allelic groups and also discriminate most four-digit alleles commonly seen in the Spanish population. All HLA typing studies were performed in a laboratory accredited by the European Federation for Immunogenetics, following the applicable quality standards issued by this organization.

**Statistical Analysis**

# Graphics and statistical analyses were performed with Graph Pad Prism 8 Software (GraphPad Software, USA, www.graphpad.com) and Stata 14.0 for Windows (Stata Corp LP, College Station, TX, USA). Quantitative variables were represented as mean ± standard deviation (SD). To analyze statistically significant differences in variables following a non-normal distribution, Mann Whitney and Kruskal-Wallis tests for unpaired samples and Wilcoxon tests for paired samples were used. For normally distributed variables, differences between groups were assessed with Student’s t-test or paired t-test, as appropriate. Qualitative variables were described as counts and proportions and 𝟀2 or Fisher´s exact test was used for comparisons. Correlation between quantitative variables following non-normal distribution was analyzed using the Spearman correlation test. Differences were considered statistically significant at P < 0.05, and individual P values are indicated in the text and/or figure legends.

**SUPPLEMENTARY FIGURE LEGENDS**

**Supplementary Figure 1. Gating strategy.** Lymphocytes were gated in the FSC/SSC dot plot, followed by singlet gating in FSC-A/FSC-H dot plot and dead cell exclusion by negative 7-AAD staining. CD4+ T cells were identified in gated cells as CD4+CD5+. Activated cells were defined as CD69+ CD25+. For comparative assays, activated cells were additionally identified as OX40+CD137+, or through positive IFNγ intracellular staining. Dot plots show staining for both SEB-activated cells and control non-stimulated lymphocytes (negative). Memory subpopulations were defined within CD69+CD25+ cells as follows: T_CM:_ CD45RO+ CD27+, T_EM_: CD45RO+ CD27-, and T_EMRA_: CD45RO- CD27-; naive CD4+ T cells were defined as CD45RO- CD27+. Specific cTfh were analyzed as CD69/CD25 double positive cells within either CD4+CXCR5+ or CD4+CXCR5+PD-1+ populations.

**Supplementary Figure 2. Comparative analysis of T cell activity. A.** Graphics show the percentage of double positive staining for CD25/CD69 or OX40/CD137, and the percentage of IFNγ-expressing CD4+ cells in either basal non-stimulated conditions (up) or after SEB-induced activation (down). Mean+SD values are shown for each condition; statistical significance was assessed by Kruskal-Wallis tests. *p<0.05; ***p<0.001. **B.** The graphic shows the absolute number of donors showing CD4+ T cell activation assessed by specific percentage of positive cells with one or more analysis strategies as indicated. **C.** Dot plots of two representative patients analyzed for T cell activity in the presence of SEB (positive control), DMSO (negative control), and peptides from Mpro and S1/S2 proteins, through parallel staining with CD25/CD69, OX40/CD137, and IFNγ. The percentage of positive cells is shown in each case. Patients show specific activation against SEB and S1 or S2, but not for Mpro. CD25: CD25/CD69; OX40: OX40/CD137. Threshold for positive activation was set as SI≥3 for CD69/CD25 and IFNγ, and ≥2 for OX40/CD137; and for specific percentage as ≥0.85% for CD69/CD25, ≥0.62% for OX40/CD137, and ≥0.3% for IFNγ.

**Supplementary Figure 3**. **T cell reactivity in healthy and convalescent donors before and after vaccination estimated as SI. A.** SARS-CoV-2-reactive T cells of HD (n=10) pre-vaccination in the presence of different peptide pools, estimated as SI. **B.** SARS-CoV-2-reactive T cells of CD (P; n=11) pre-vaccination in the presence of different peptide pools, estimated as SI. **C.** SARS-CoV-2-reactive T cells of vaccinated HD (left) and CD (right) after stimulation with different peptide pools, estimated as SI. SI was considered positive when ≥3 (dotted lines). **D.** Comparison of CD4+ T memory subsets specific for SARS-CoV-2 peptide pools in vaccinated HD and CD; ** p≤0.01; ns, non-significant. **E.** Correlation between SARS-CoV-2 S1 specific CD4+ T cells and SARS-CoV-2 S2 specific CD4+ T cells (%) in CD pre-vaccination. Spearman correlations were calculated. Fitted linear prediction and its 95% confidence interval (transparent gray shadow) are shown.

**Supplementary Figure 4. Comparison of antibody levels between healthy and convalescent donors after vaccination. A.** Antibody profile in HD and CD before vaccine administration. Plates coated with Mpro, NP, S and RBD were used to perform ELISA tests. Graphs represent the mean and standard deviation of all samples tested. **B.** Detection of IgG, IgA and IgM directed against the Mpro, S and RBD in vaccinated donors. Whisker and box plots of 23 healthy and 25 CD are shown. Sera dilutions of 1/3200 for IgG anti-S and anti-RBD, 1/200 for IgG anti-MPro, and 1/50 for IgA and IgM were used. In all cases, optical density at 492 nm was normalized using the signal obtained with a pool of positive sera, and statistical significance was assessed by means of t-test or Mann-Whitney test when groups followed normal or non-normal distribution, respectively. Positivity threshold was calculated as 3 SD above mean in healthy donors (dotted line). * p<0.05, **p<0.01, **** p<0.0001.

**Supplementary Figure 5. Circulating Tfh in vaccinated healthy and convalescent donors.** Percentage of SARS-CoV-2-specific CD4+CXCR5+ (**A.**) and CD4+CXCR5+PD-1hi cTfh cells (**B.**) in healthy and convalescent donors after vaccination. In both cases, graphics show total percentage of specific cTfh (left), S1-responsive cTfh (middle), and S2-responsive cTfh (right) cells. ns, non-significant.

**SUPPLEMENTARY TABLE 1**

|  | **Convalescent individuals**  **n=23** | **Healthy donors**  **n=22** |
| --- | --- | --- |
| **Tested for antibodies and T cell memory** | 11 | 10 |
| **Tested only for antibodies** | 12 | 12 |
| **Sex (male)** | 10 (43.5) | 6 (27.3) |
| **Age (years)** | 55 (40-58) | 52 (34-60) |
| **Exact date of inclusion in the study (mm/dd/yy)** | 07/06/2020 | 04/14/2020 |
| **Antibody assay pre-vaccination (days POS)** | 114 (107-119) | _ |
| **T cell assay pre-vaccination (days POS)** | 155 (129-194) | _ |
| **Antibody assay post-vaccination (days after second dose) administration)** | 40 (36-41) | 41.5 (39-42) |
| **T cell assay post-vaccination (days after second dose) administration)** | 62 (52-70) | 62 (49-69) |
| **Symptoms** | 22 (95.7) | _ |
| **Fever** | 18 (78.2) | _ |
| **Headache** | 11 (47.8) | _ |
| **Anosmia** | 8 (34.8) | _ |
| **Dry cough** | 11 (47.8) | _ |
| **Dyspnea** | 7 (30.4) | _ |
| **Hospitalization** | 2 (8.7) | _ |
| **Laboratory findings at baseline** |  |  |
| **D-dimer (µg/mL)** | 0.33 (0.27-0.42) | 0.31 (0.25-0.39) |
| **LDH (U/L)** | 170 (165.25-181.5) | 156 (152.5-171.75) |
| **CRP (mg/dL)** | 0.06 (0.05-0.08) | 0.11 (0.06-0.19) |
| **IgG (mg/dL)** | 1045 (888.5-1177.5) | 1090 (993-1232.5) |
| **IgA (mg/dL)** | 215 (155.5-291.25) | 229 (173.5-279.5) |
| **IgM (mg/dL)** | 109 (94.45-178.5) | 121 (100.75-207.25) |
| **C3 (mg/dL)** | 94.8 (84.6-105.25) | 91 (79.4-101.88) |
| **C4 (mg/dL)** | 19.65 (16.5-25.3) | 19.5 (14.85-25.75) |
| **IL-6 (pg/mL)** | 0.2 (0.2-2.25) | 2 (1-42.75) |
| **Leukocytes x10^9^/L** | 5.9 (5.3-6.96) | 6.3 (5.3-6.95) |
| **Lymphocytes x10^9^/L** | 1.8 (1.6-2.3) | 2.25 (1.97-2.8) |
| **Treatment** | 9 (39.1) | _ |
| **Hydroxychloroquine** | 8 (34.8) | _ |
| **Glucocorticoids** | 2 (8.7) | _ |
| **Tocilizumab** | 1 (4.3) | _ |
| **Antibiotic therapy** | 5 (21.7) | _ |
| **Oxigen therapy** | 2 (8.7) | _ |
| **Comorbidities** | 9 (39.1) | 4 (18.2) |
| **HBP** | 5 (21.7) | 1 (4.5) |
| **DM** | 2 (8.7) | 0 (0) |
| **COPD** | 2 (8.7) | 0 (0) |
| **DL** | 0 (0) | 3 (13.6) |
| **Asthma** | 2 (8.7) | 0 (0) |
| **Hypothyroidism** | 1 (4.3) | 2 (9.1) |
| **Autoimmune disease** | 0 (0) | 2 (9.1) |
| **Disease Severity Degree*** |  |  |
| **1** | 13 | _ |
| **2** | 9 | _ |
| **3** | 1 | _ |

POS: post onset of symptoms, LDH: lactate dehydrogenase, CRP: C reactive protein, HBP: high blood pressure, DM: diabetes mellitus, COPD: chronic obstructive pulmonary disease, DL: dyslipidemia.

**Supplementary Table 1. Demographic and clinical characteristics of the study population**. All categorical variables are expressed as number (%) and quantitative variables as median (IQR)

*(5)

**SUPPLEMENTARY TABLE 2**

| **DONOR** | **HLA-DRB1** | | **CD4+ PRE-VACCINATION** | **CD4+ POST-VACCINATION** |
| --- | --- | --- | --- | --- |
| **HD1** | **11:01** | **13:01** | **S1 S2 VME1 NCAP** | **S1 S2** |
| **HD2** | **04:04** | **11:03** | **S1 S2** | **RBD S1 S2** |
| **HD3** | **03:01** | **07:01** | **-** | **S1 S2** |
| **HD4** | **03:01** | **13:01** | **NCAP** | **S2** |
| **HD5** | **04:04** | **08:01** | **-** | **S2** |
| **HD6** | **11:04** | **13:02** | **-** | **S1 S2** |
| **HD7** | **07:01** | **11:01** | **-** | **S1** |
| **HD8** | **01:01** | **10:01** | **-** | **S1 S2** |
| **HD9** | **01:02** | **13:01** | **-** | **S1** |
| **HD10** | **07:01** | **15:01** | **S1 NCAP** | **S1 S2** |
| **P1** | **07:01** | **13:03** | **S1** | **S1 S2 RBD** |
| **P2** | **01:02** | **03:01** | **S1** | **S1 S2** |
| **P4** | **01:03** | **15:01** | **S1** | **S1 S2** |
| **P5** | **15:01** | **15:01** | **-** | **-** |
| **P6** | **07:01** | **15:01** | **S1 VME1** | **S1 S2 VME1** |
| **P7** | **14:02** | **15:01** | **NCAP VME1** | **S1 S2 VME1** |
| **P9** | **04:02** | **08:02** | **-** | **S1 S2** |
| **P10** | **03:01** | **07:01** | **-** | **-** |
| **P11** | **04:03** | **04:04** | **-** | **S1 S2 RBD** |

**Supplementary Table 2. Class II HLA-DRB1 genotype in healthy and convalescent donors**. HLA-DRB1 genotype and pre/post-vaccination CD4+ specificities are shown for healthy (HD) and convalescent (P) donors.

**SUPPLEMENTARY REFERENCES**

1. El Hentati FZ, Gruy F, Iobagiu C, Lambert C. Variability of CD3 membrane expression and T cell activation capacity. Cytometry B Clin Cytom. 2010;78(2):105-14.

2. Durrieu F, Geneviève F, Arnoulet C, Brumpt C, Capiod JC, Degenne M, et al. Normal levels of peripheral CD19(+) CD5(+) CLL-like cells: toward a defined threshold for CLL follow-up -- a GEIL-GOELAMS study. Cytometry B Clin Cytom. 2011;80(6):346-53.

3. Grifoni A, Weiskopf D, Ramirez SI, Mateus J, Dan JM, Moderbacher CR, et al. Targets of T Cell Responses to SARS-CoV-2 Coronavirus in Humans with COVID-19 Disease and Unexposed Individuals. Cell. 2020;181(7):1489-501.e15.

4. Martínez-Fleta P, Alfranca A, González-Álvaro I, Casasnovas JM, Fernández-Soto D, Esteso G, et al. SARS-CoV-2 Cysteine-like Protease Antibodies Can Be Detected in Serum and Saliva of COVID-19-Seropositive Individuals. J Immunol. 2020;205(11):3130-40.

5. Wu Z, McGoogan JM. Characteristics of and Important Lessons From the Coronavirus Disease 2019 (COVID-19) Outbreak in China: Summary of a Report of 72 314 Cases From the Chinese Center for Disease Control and Prevention. Jama. 2020;323(13):1239-42.
